# Sox13 is a novel flow-sensitive transcription factor that prevents inflammation by repressing chemokine expression in endothelial cells

**DOI:** 10.3389/fcvm.2022.979745

**Published:** 2022-09-30

**Authors:** Catherine Demos, Janie Johnson, Aitor Andueza, Christian Park, Yerin Kim, Nicolas Villa-Roel, Dong-Won Kang, Sandeep Kumar, Hanjoong Jo

**Affiliations:** ^1^Wallace H. Coulter Department of Biomedical Engineering, Georgia Institute of Technology, Emory University, Atlanta, GA, United States; ^2^Division of Cardiology, Department of Medicine, Emory University, Atlanta, GA, United States

**Keywords:** shear-sensitive TF, Sox13, CCL5, CXCL10, endothelium, inflammation

## Abstract

Atherosclerosis is a chronic inflammatory disease and occurs preferentially in arterial regions exposed to disturbed blood flow (d-flow) while the stable flow (s-flow) regions are spared. D-flow induces endothelial inflammation and atherosclerosis by regulating endothelial gene expression partly through the flow-sensitive transcription factors (FSTFs). Most FSTFs, including the well-known Kruppel-like factors KLF2 and KLF4, have been identified from *in vitro* studies using cultured endothelial cells (ECs). Since many flow-sensitive genes and pathways are lost or dysregulated in ECs during culture, we hypothesized that many important FSTFs in ECs *in vivo* have not been identified. We tested the hypothesis by analyzing our recent gene array and single-cell RNA sequencing (scRNAseq) and chromatin accessibility sequencing (scATACseq) datasets generated using the mouse partial carotid ligation model. From the analyses, we identified 30 FSTFs, including the expected *KLF2/4* and novel FSTFs. They were further validated in mouse arteries *in vivo* and cultured human aortic ECs (HAECs). These results revealed 8 FSTFs, *SOX4, SOX13, SIX2, ZBTB46, CEBPβ, NFIL3, KLF2*, and *KLF4*, that are conserved in mice and humans *in vivo* and *in vitro*. We selected *SOX13* for further studies because of its robust flow-sensitive regulation, preferential expression in ECs, and unknown flow-dependent function. We found that siRNA-mediated knockdown of SOX13 increased endothelial inflammatory responses even under the unidirectional laminar shear stress (ULS, mimicking s-flow) condition. To understand the underlying mechanisms, we conducted an RNAseq study in HAECs treated with SOX13 siRNA under shear conditions (ULS vs. oscillatory shear mimicking d-flow). We found 94 downregulated and 40 upregulated genes that changed in a shear- and SOX13-dependent manner. Several cytokines, including *CXCL10* and *CCL5*, were the most strongly upregulated genes in HAECs treated with SOX13 siRNA. The robust induction of CXCL10 and CCL5 was further validated by qPCR and ELISA in HAECs. Moreover, the treatment of HAECs with Met-CCL5, a specific CCL5 receptor antagonist, prevented the endothelial inflammation responses induced by siSOX13. In addition, SOX13 overexpression prevented the endothelial inflammation responses. In summary, SOX13 is a novel conserved FSTF, which represses the expression of pro-inflammatory chemokines in ECs under s-flow. Reduction of endothelial SOX13 triggers chemokine expression and inflammatory responses, a major proatherogenic pathway.

## Introduction

Atherosclerosis is a leading cause of death worldwide as the major underlying cause of myocardial infarction, stroke, and peripheral arterial disease (1). Atherosclerotic plaques preferentially develop in curved or branched arterial regions exposed to disturbed flow (d-flow), characterized by low and oscillatory shear stress (OSS). In contrast, artery regions exposed to stable flow (s-flow) with characteristic unidirectional, high laminar shear stress (ULS) are protected from the disease (2–6). D-flow and s-flow are recognized by mechanosensors, which trigger a wide spectrum of different endothelial responses. While s-flow/ULS promotes a healthy endothelial phenotype, d-flow/OSS induces atherosclerosis development and progression (7–11) by regulating gene expression on a genome-wide scale (9, 12–17). Transcription factors regulated in a flow-sensitive manner (FSTFs) play a key role in genome-wide gene expression by driving transcription of flow-sensitive genes (9, 10, 13, 15, 18).

Many FSTFs, such as Kruppel-like factors (KLF2 and KLF4), are well-known to regulate the expression of hundreds of target genes and numerous anti- or pro-atherogenic pathways (14, 19–23). KLF2 overexpression in static ECs induces key flow-sensitive pathways, demonstrating the importance of FSTFs in ECs (24). Other FSTFs include NF-κB, AP-1, and YAP/TAZ, which regulate inflammatory signaling, proliferation, and migration in response to d-flow/OSS in endothelial cells (ECs) (25–29). These well-known FSTFs have been mostly identified from ECs cultured *in vitro* (26), while similar *in vivo* studies are lacking. Since ∼45% of flow-sensitive genes identified *in vivo* are estimated to be dysregulated or lost in ECs during culture *in vitro* (17, 30), we hypothesized that some important, highly sensitive FSTFs active under *in vivo* conditions had yet to be discovered.

**FIGURE 1:**
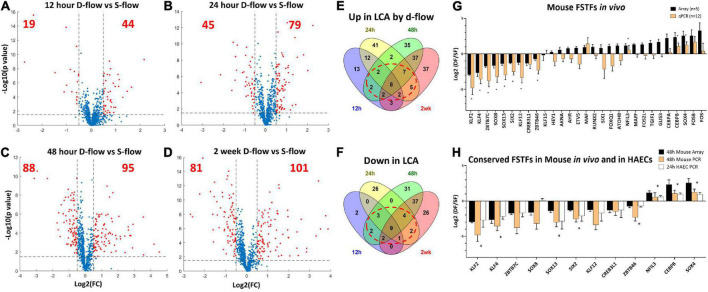
Identification of flow-sensitive transcription factors (FSTFs) in mouse artery ECs *in vivo* and validation *in vivo* and *in vitro*. Volcano plots show differentially expressed TFs in endothelial-enriched RNAs in the LCAs (left carotid artery exposed to d-flow) and RCAs (right carotid artery exposed to s-flow) obtained at **(A)** 12 h, **(B)** 24 h, **(C)** 48 h, and **(D)** 2 weeks after the PCL in mice. Red dots indicate TFs changed ≥50% with a *p*-value ≤ 0.05. **(E,F)** Venn diagrams show TFs commonly upregulated or downregulated by d-flow at 3 or 4 different time points. **(G)** qPCR validation of 29 potential FSTFs at 48 h post-PCL. Mean fold change ±SEM, *n* = 12 for qPCR, (*) indicates *p* ≤ 0.05 for qPCR results calculated by student’s *t*-test. **(H)** The *in vivo* gene array and qPCR data at 48 h post-PCL results are compared to the *in vitro* qPCR results in HAECs exposed to oscillatory shear (OSS mimicking d-flow) and unidirectional laminar shear (ULS, mimicking s-flow). Mean fold change ±SEM, *n* = 5 for HAECs, (*) indicates *p* < 0.05 for 24 h HAEC qPCR calculated by student’s *t*-test.

To identify novel FSTFs in artery ECs *in vivo*, we re-analyzed two independent datasets that we previously reported using our mouse partial carotid ligation (PCL) model, the d-flow-induced mouse model of atherosclerosis (12, 13). In the PCL model (Figure 1), three of the four caudal branches (left external carotid, internal carotid, and occipital artery) of the left common carotid artery (LCA) are ligated, leaving the superior thyroid artery patent. In the same animal, the contralateral right carotid artery (RCA) with characteristic ULS continues to be exposed to s-flow and serves as a control. The PCL causes *d-flow* with characteristic OSS in the LCA, rapidly inducing robust atherosclerosis within 2 weeks, directly demonstrating a causal relationship between d-flow and atherosclerosis (31, 32). To understand the mechanisms of flow-induced atherosclerosis, we previously carried out a gene array study using endothelial-enriched “bulk” RNAs from the LCAs and RCAs obtained at four-time points (12 h, 24 h, 48 h, and 2 weeks) after the PCL surgery (12). In addition, we performed a single-cell RNA sequencing (scRNAseq) and single-cell Assay for Transposase-Accessible Chromatin with high-throughput sequencing (scATACseq) using the LCA and RCA RNAs obtained at 2-day and 2-week post-PCL time points (13). The scRNAseq and scATACseq studies showed that d-flow induces genome- and epigenome-wide changes in gene expression, reprogramming endothelial cells from the healthy atheroprotective phenotype to pro-inflammatory and pro-atherogenic phenotypes, including the transition of ECs to mesenchymal (EndMT) and immune cell-like (EndICLT) cells (13).

Here, we re-analyzed the *in vivo* datasets from the PCL studies (gene array, scRNAseq, and scATACseq datasets) and found several novel FSTFs, including SOX13, that are conserved in mouse artery ECs *in vivo* and human aortic ECs (HAECs) *in vitro*. SRY (Sex Determining Region Y)-box transcription factor 13 (SOX13) is a member of the SoxD subfamily of the SRY-related high mobility group (HMG) box (Sox) transcription factors and is involved in the regulation of embryonic development and the determination of cell fate (33). SOX13 is also known as islet cell antibody 12 (ICA12), as it is a type-1 diabetes autoantigen. Critically, SOX13 binds with TCF1 competitively to β-catenin, thereby indirectly preventing TCF1/ β-catenin targets from being transcribed in embryonic tissues (34). SOX13 has also been studied in various cancers where many functions are related to stem cell fate determination (35–38). SOX13 protein expression is high in embryonic arterial walls, suggesting part of the developmental role is in arteriogenesis (39, 40). SOX13 flow sensitivity has not previously been reported, nor has a role in atherosclerosis emerged. To examine SOX13 functional effects and transcriptional targets under flow, we further conducted RNAseq and functional studies using HAECs treated with SOX13 siRNA (siSOX13) or overexpression plasmid under shear conditions. The results show that SOX13 is a novel FSTF induced by s-flow and represses the expression of cytokines CCL5 and CXCL10, preventing endothelial inflammation. The loss of SOX13, in contrast, dramatically induces the expression of the cytokines, leading to robust endothelial inflammation.

## Results

### Identification and validation of novel factors regulated in a flow-sensitive manner in mouse artery endothelial cells *in vivo* and in cultured human aortic endothelial cells

To identify FSTFs in mouse artery ECs *in vivo*, we first re-analyzed our genome-wide transcriptome data using the EC-enriched bulk RNAs from the mouse PCL study (12). The gene array dataset (GSE182291) was comprised of transcript expression results of LCAs (exposed to d-flow) and RCAs (contralateral s-flow) obtained at four-time points: 12 h, 24 h, 48 h, and 2 weeks post-PCL. We first cross-checked the list of human TFs (41) to our mouse gene array data and found 973 TFs detected at least at the one-time point. Then, we determined differential expression in LCA vs. RCA at each time point to identify TFs that changed by flow by ≥50% with a *p*-value ≤ 0.05, representing our definition of FSTFs. The total number of FSTFs at each time point was 63 at 12 h, 124 at 24 h, 183 at 48 h, and 182 at 2 weeks, totaling 323 unique FSTFs changing at least at one time-point (Figures 1A–D). Of the 323, 8 upregulated and 9 downregulated FSTFs changed in the consistent direction by *d-flow* at all four time-points (Figures 1E,F). In addition, we found 20 upregulated and 14 downregulated TFs that changed consistently in three time points. Of those, we removed any TFs that changed in the opposite direction at any time point. Through the analyses, we found 30 FSTFs that changed consistently in one direction, up or down, at more than 3 time points in mouse artery ECs.

Next, we validated the gene array data of these 30 potential FSTFs by qPCR using additional RNA samples obtained from LCAs and RCAs at 48 h post-PCL (Figure 1G). We confirmed the flow sensitivity of 12 of 29 potential FSTFs, while one (*TCF23*) was not detectable by qPCR. Next, we examined mRNA expression in HAECs to determine if the mouse FSTFs were also conserved in human ECs and flow-sensitive *in vitro*. In HAECs subjected to ULS (mimicking s-flow) or OSS (mimicking d-flow) for 24 h, we validated the expression of 8 of the 12 TFs that were regulated by flow in a consistent manner (Figure 1H). Five upregulated FSTFs by ULS were *KLF2, KLF4, SOX13, SIX2*, and *ZBTB46*, and 3 upregulated by OSS were *NFIL3, CEBPβ*, and *SOX4* (Figure 1H). *KLF2, KLF4, ZBTB46*, and *SOX4* (42–45) were previously shown to be FSTFs in ECs, demonstrating the validity of our approach. In contrast, *SOX13, SIX2, CEBPβ, and NFIL3* are novel potential FSTFs.

### Validation of Sox13 as a flow-sensitive transcription factor by re-analyzing the *in vivo* single-cell RNA sequencing and single-cell assay for transposase-accessible chromatin with high-throughput sequencing datasets of mouse artery endothelial cells

**FIGURE 2:**
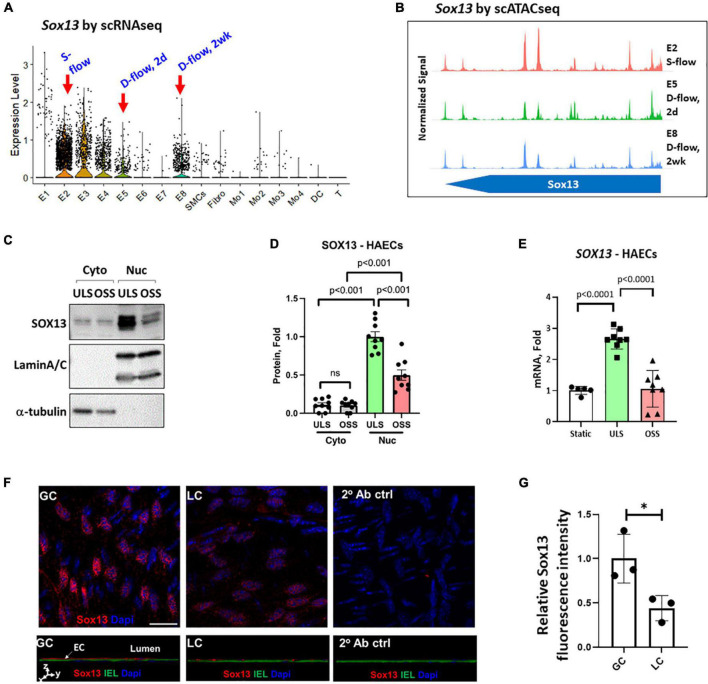
Validation of SOX13 flow sensitivity in ECs. **(A)** Violin plot shows *SOX13* expression profile in our published scRNAseq data obtained from the mouse LCAs and RCAs at the 2-day (acute) or 2 weeks (chronic) post-PCL (partial carotid ligation) surgery. E2 represents a healthy EC cluster exposed to s-flow in RCA. E5 and E8 are EC clusters exposed to acute and chronic d-flow, respectively. **(B)** Chromatin accessibility plot of *Sox13* gene was obtained by re-analyzing the published scATACseq data using the same mouse PCL model. E2 (s-flow), E5 (acute d-flow), and E8 (chronic d-flow) show a time-dependent closure of SOX13 accessibility by d-flow in ECs. **(C–E)** HAECs exposed to ULS or OSS for 24 h were lysed, fractionated into the cytoplasmic (cyto) and nuclear (nuc) fractionations, and western blot analyzed using antibodies to SOX13, laminA/C, and α-tubulin **(C)** and quantified **(D)**. Mean±SEM, *n* = 5, *p*-values calculated by student’s *t*-test. **(E)** Shows *SOX13* qPCR from a similar HAEC shear study, Mean±SEM, *n* = 8, *p*-values calculated by student’s *t*-test. **(F)** Shows immunofluorescence staining of Sox13 of mouse GC (greater curvature exposed to stable flow) and LC (lesser curvature exposed to disturbed flow) naturally in the aortic arch. The bottom panels show orthogonal views, showing SOX13 expression above the internal elastic lamina (IEL). DAPI shows nuclei. **(G)** Shows the quantitation of SOX13 expression. Mean±SEM, *n* = 3 mice, *p* ≤ 0.05 by student’s *t*-test.

We also re-analyzed our published scRNAseq data from the mouse PCL study to further validate the flow sensitivity of *Sox13, Six2, Cebpβ*, and *Nfil3*. Four groups were represented in this study: RCA/s-flow or LCA/d-flow for 2 days or 2 weeks each. *Sox13* showed a strong flow sensitivity and preferential expression in ECs compared to other artery wall cell types (smooth muscle cells, immune cells, and fibroblasts) (Figure 2A). Briefly, the E1 cluster consisted of ECs present in all 4 conditions (2d-RCA, 2d-LCA, 2wk-RCA, and 2wk-LCA). E1–E4 consisted of ECs exposed to s-flow conditions (2d–RCA and 2wk–RCA). The majority of E5 and E7 clusters consisted of ECs exposed to acute d-flow (2d-LCA). E6 and E8 exclusively consisted of ECs exposed to chronic d-flow (2wk-LCA). *Sox13* expression was significantly higher in the healthy EC subpopulation (E2) found in s-flow-exposed RCAs compared to the pro-atherogenic, *d-flow*-exposed EC subpopulations (E5 and E8) (13). Although E5 (acute d-flow) expressed lower Sox13 levels than E8 (chronic d-flow), both ECs showed significantly lower levels compared to E2 (s-flow condition). We also re-analyzed the scATACseq data and found that *Sox13* chromatin accessibility was more open (indicating higher gene transcription activity) in the healthy E2 under the s-flow condition compared to the pro-atherogenic E5 and E8 populations under the acute (2-day) and chronic (2-week) d-flow conditions, respectively (Figure 2B and Supplementary Table 1). The scATACseq and the scRNAseq data analyses further support the mouse gene array data and HAEC validation results (Figure 1), demonstrating that *Sox13* is indeed a novel FSTF in ECs. Therefore, we decided to focus on studying SOX13 further in this study.

### Stable flow induces SOX13 expression in the nucleus of human aortic endothelial cells and in mouse aortic endothelial cells

We examined the effect of shear on SOX13 protein expression in the cytoplasmic and nuclear fractions in HAECs. SOX13 protein was predominantly expressed in the nucleus, and exposure to ULS significantly increased the expression in the nuclear fraction compared to the static or OSS conditions (Figures 2C,D). ULS exposure also significantly increased *SOX13* mRNA expression compared to OSS and static conditions in HAECs (Figure 2E). These data further demonstrated that SOX13 protein is primarily expressed in the nucleus, as expected for a TF, under *s-flow* conditions in HAECs.

Furthermore, we examined Sox13 protein in mouse aortic arch regions in an immunofluorescence staining study using the greater curvature (GC) exposed to stable flow and lesser curvature (LC) exposed to disturbed flow intrinsically. The staining result (Figures 2F,G) clearly demonstrated the specific staining of Sox13 above the internal elastic layer (IEL), demonstrating a higher SOX13 expression in the endothelial layer in the aorta naturally exposed to stable flow in GC than disturbed flow in LC region. This result further supports that Sox13 protein expression is higher in the naturally stable flow region than the unstable flow region in mouse aorta *in vivo*.

### SOX13 knockdown induces cytokine and chemokine expression in human aortic endothelial cells

**FIGURE 3:**
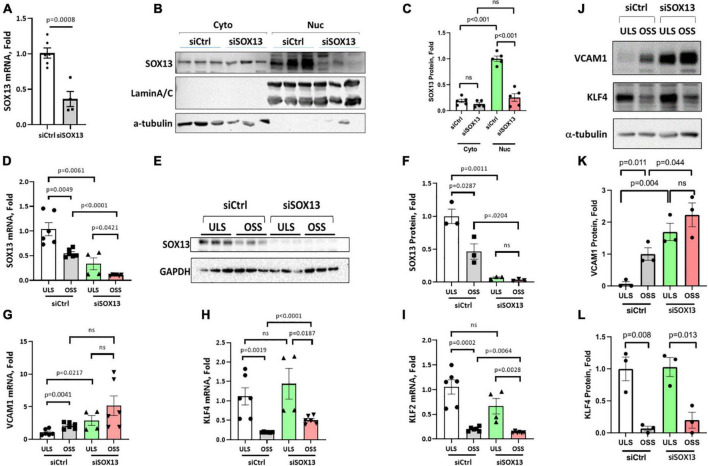
siSOX13 knockdown of SOX13 in HAECs. **(A–C)** Static HAEC study. HAECs were treated with siSOX13 vs. siCtrl for 48 h, and analyzed by qPCR **(A)** and western blot using cytoplasmic and nuclear fractions with SOX13 antibody **(B)** and quantified **(C)**. **(D–L)** Shear study. HAECs treated with siSOX13 vs. siCtrl for 48 h were exposed to ULS or OSS for another 24 h, and analyzed for SOX13 by qPCR **(D)**, western blot of whole cell lysate **(E,F)**. Additional qPCR assays for *VCAM1*, *KLF4*, and *KLF2* **(G–I)** and western blots for VCAM1 and KLF4 **(J,L)** were conducted. Mean±SEM, *n* = 4–6 **(F,I)** or 3 **(K,L)** are shown, and *p*-values calculated by student’s *t*-test.

To determine how SOX13 regulates EC function, we first knocked down SOX13 with two pooled siRNAs to SOX13 (siSOX13). Treatment of HAECs with the siSOX13 effectively reduced *SOX13* mRNA (Figure 3A) and nuclear SOX13 protein levels under the static or flow conditions (Figures 3B–F). As controls, we measured the shear-dependent expression of *KLF2*, *KLF4*, and *VCAM1* under the ULS and OSS conditions in HAECs treated with siSOX13 and control siRNA (siCtrl) (Figures 3G–L). As expected, the ULS exposure decreased the expression of *VCAM1* while increasing *KLF2* and *KLF4* compared to the OSS condition. Interestingly, we found that *VCAM1* mRNA and protein expression was increased by siSOX13 compared to siCtrl, indicating potential endothelial inflammation even under the ULS condition. In contrast, *KLF2* and *KLF4* did not significantly change by the siSOX13 treatment under the ULS condition.

To explore the underlying mechanism of SOX13 knockdown effect, we next conducted RNA sequencing (RNAseq) analysis in four groups of HAECs treated with siSOX13 or siCtrl and ULS or OSS conditions: (1) siCtrl ULS, (2) siSOX13 ULS, (3) siCtrl OSS, and (4) siSOX13 OSS. HAECs were transfected with siSOX13 or siCtrl for 24 h, then subjected to ULS or OSS for an additional 24 h, and the total RNA was analyzed by RNAseq (*n* = 4). To identify the genes (GSE207087) regulated by a flow- and siSOX13-dependent manner, we screened for differentially expressed genes that changed by ≥50% with a *p*-value of ≤0.05 between the four groups. The gene list was further filtered to remove low-abundance genes with ≤50 mean total counts/sample in all groups.

**FIGURE 4:**
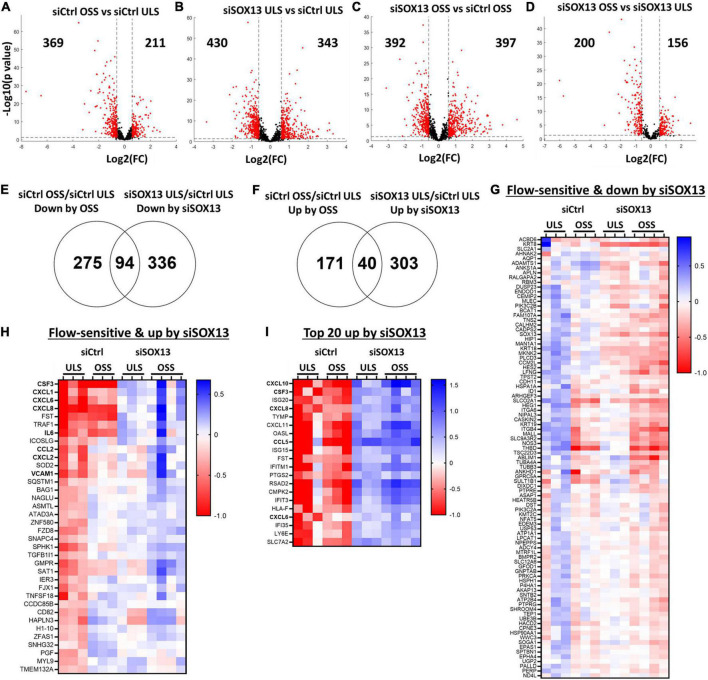
Shear-dependent and SOX13-dependent gene expression in HAECs by RNAseq analysis. HAECs treated with siSOX13 or siCtrl for 24 h were exposed to OSS or ULS for another 24 h, and total RNAs were analyzed by RNAseq. **(A–D)** Shows differential gene expression by comparing the four groups: (1) siCtrl OSS, (2) siSOX13 OSS, (3) siCtrl LSS, and (4) siSOX13 LSS. The number of significantly downregulated (left) or upregulated (right) genes by ≥50% with a *p*-value ≤ 0.05 (shown as red dots) are indicated in each volcano plot. **(E,F)** Venn diagrams show commonly downregulated genes by OSS or siSOX13 **(E)** and upregulated genes by OSS or siSOX13 in ULS condition **(F)**. **(G,H)** Heat map of the 94 commonly downregulated genes by OSS or siSOX13 in ULS condition **(G)** and the 40 commonly upregulated by OSS or by siSOX13. **(I)** Heatmap of top 20 genes upregulated by siSOX13 regardless of shear conditions.

First, a comparison between the siCtrl OSS and siCtrl ULS showed 369 downregulated and 211 upregulated genes by OSS under the basal (siCtrl) condition (Figure 4A). Next, we identified SOX13-dependent genes under the ULS condition. siSOX13 treatment under ULS condition downregulated 430 genes while upregulating 343 genes compared to the siCtrl ULS (Figure 4B), demonstrating the major impact of SOX13 knockdown on ULS-dependent gene expression. siSOX13 treatment under the OSS condition also downregulated 392 and upregulated 397 genes compared to the siCtrl OSS (Figure 4C), demonstrating the robust effect of SOX13 knockdown in this acute OSS condition. The comparison between siSOX13 OSS and siSOX13 ULS showed 200 upregulated and 156 downregulated genes (Figure 4D).

We next examined which genes were commonly upregulated or downregulated by OSS (vs. ULS) or siSOX13 (vs. siCtrl) conditions in HAECs by comparing the differentially expressed gene lists as shown in the Venn diagrams (Figures 4E,F). The comparison revealed 94 genes commonly downregulated by OSS (siCtrl OSS vs. siCtrl ULS) or siSOX13 under ULS conditions (siSOX13 ULS vs. siCtrl ULS) (Figure 4E). This 94 gene list (Figure 4G) contained *SOX13* (as expected by siSOX13 treatment), *AQP1* (a predicted SOX13 gene target) (46), *THBD*, and *NOS3.* We next found 40 genes commonly upregulated by OSS (siCtrl OSS vs. siCtrl ULS) or siSOX13 under ULS conditions (siSOX13 ULS vs. siCtrl ULS) (Figure 4F). Interestingly, the 40 gene list (Figure 4H) included many cytokines and chemokines, *CXCL1, CXCL2, CXCL6, CXCL8, CSF3, IL6*, and *CCL2*. In addition, we found the top 20 most upregulated genes by SOX13 knockdown regardless of the shear conditions. The 20 gene list contained additional cytokines and chemokines, including *CCL5* and *CXCL10* (Figure 4I). These results suggest that SOX13 is an FSTF regulating cytokine and chemokine expression in ECs.

### Flow- and SOX13-dependent biological processes

**FIGURE 5:**
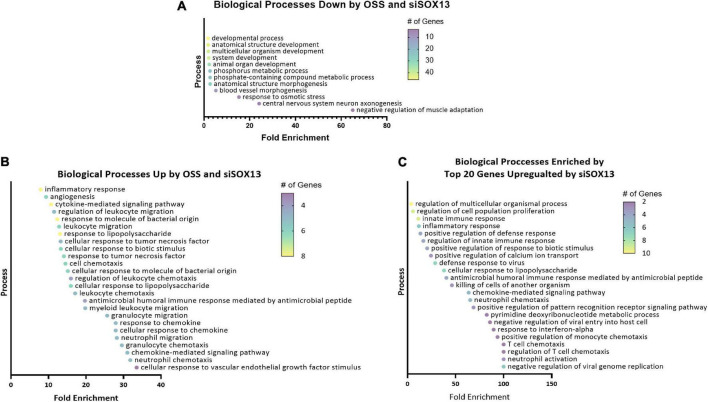
Gene ontology analysis of shear-dependent and SOX13-dependent genes. Biological processes enriched by the 94 commonly downregulated genes by OSS or siSOX13 in ULS condition **(A)**, the 40 commonly upregulated by OSS or by siSOX13 **(B)**, and the top 20 genes upregulated by siSOX13 regardless of shear conditions **(C)** are shown. All biological processes listed were significant by *p*-value.

To predict the role of SOX13 and its target genes in endothelial function and atherosclerosis, we conducted a gene ontology (GO) analysis using the commonly regulated gene lists (Figures 4G,I). The GO analysis of the 94 commonly downregulated genes by OSS or siSOX13 under the ULS condition (Figure 4H) showed that biological processes related to development, anatomical structure, blood vessel morphogenesis, and cell fate were enriched (Figure 5A), known SOX13-dependent processes (33, 39, 47, 48). In contrast, the GO analysis of the 40 genes upregulated by OSS and SOX13 knockdown under ULS conditions (Figure 4I) showed enrichment of 25 biological processes (Figure 5B). Interestingly, 17 of the 25 enriched biological processes were related to inflammation, including inflammatory response, cytokine-mediated signaling, response to chemokine leukocyte migration, and leukocyte chemotaxis. In addition, the GO analysis of the top 20 most upregulated genes by the siSOX13 treatment, regardless of the shear conditions, revealed the predominant enrichment of immune cell infiltration processes, including migration and chemotaxis of monocytes and T-cells (Figure 5C). These results strongly suggest that the reduction of SOX13 by either OSS or siSOX13 triggers a strong pro-inflammatory response, a d-flow-induced and pro-atherogenic pathway (49–53), by inducing the expression of cytokines and chemokines in ECs.

### SOX13 regulates pro-inflammatory chemokine expression in endothelial cells

**FIGURE 6:**
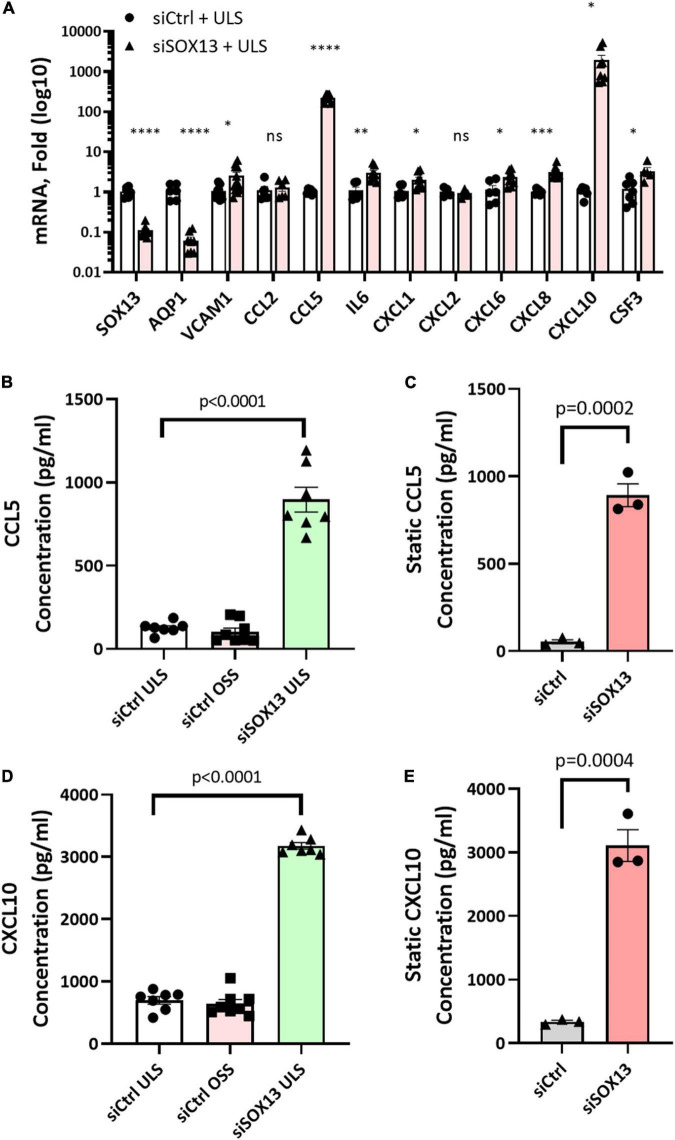
Validation of SOX13 targets by qPCR and ELISA. HAECs treated with siSOX13 or siCtrl for 24 h were exposed to OSS or ULS for another 24 h, and total RNAs were analyzed by RNAseq to validate the top SOX13-dependent targets **(A)** *n* = 4–7, Mean±SEM are shown. *p ≤ 0.05, **p ≤ 0.01 ***p ≤ 0.001, and ****p ≤ 0.0001 as determined by student’s *t*-test. The conditioned media from HAECs treated with siSOX13 and shear **(B,D)** or static **(C,E)** conditions were analyzed by ELISA quantification of CCL5 **(B,C)** or CCL10 **(D,E)**. Mean±SEM are shown, *p*-values determined by student’s *t*-test.

Our heat map and GO analyses (Figures 4, 5) showed that loss of SOX13 strongly induced pro-inflammatory chemokines and cytokines. To validate the results, we conducted a qPCR analysis on 10 of the most interesting potential targets identified from the analyses, using *SOX13* and *AQP1* as positive controls (Figure 6A). We found that treatment with siSOX13 under the ULS condition induced expression of *CXCL1, CXCL6, CXCL8 (IL8), CXCL10, IL6, CCL2 (MCP1)*, *CCL5*, and *CSF3* in HAECs. Interestingly, *CCL5* and *CXCL10* mRNAs were two of the most dramatically upregulated chemokines by siSOX13.

Although CCL5 and CXCL10 expression did not change significantly in HAECs in response to LSS or OSS for a 24 h period, *in vitro*, they rapidly responded to SOX13 knockdown. Upon analysis of our scRNAseq and gene array data, both CCL5 and CXCL10 expression increase dramatically at the longer term, 2 week time point (Supplementary Figure 1). Flow-dependent expression does not occur *in vivo* in the 2 day and shorter time points, consistent with the short-term 24 h *in vitro* shear results.

To further validate chemokine expression due to SOX13 knockdown, we measured CCL5 and CXCL10 protein levels by ELISA in the conditioned media (CM). Under both the static and shear conditions, siSOX13 treatment dramatically induced the expression of CCL5 and CXCL10 compared to siCtrl, further confirming the SOX13 knockdown effect on these chemokines (Figures 6B–E).

**FIGURE 7:**
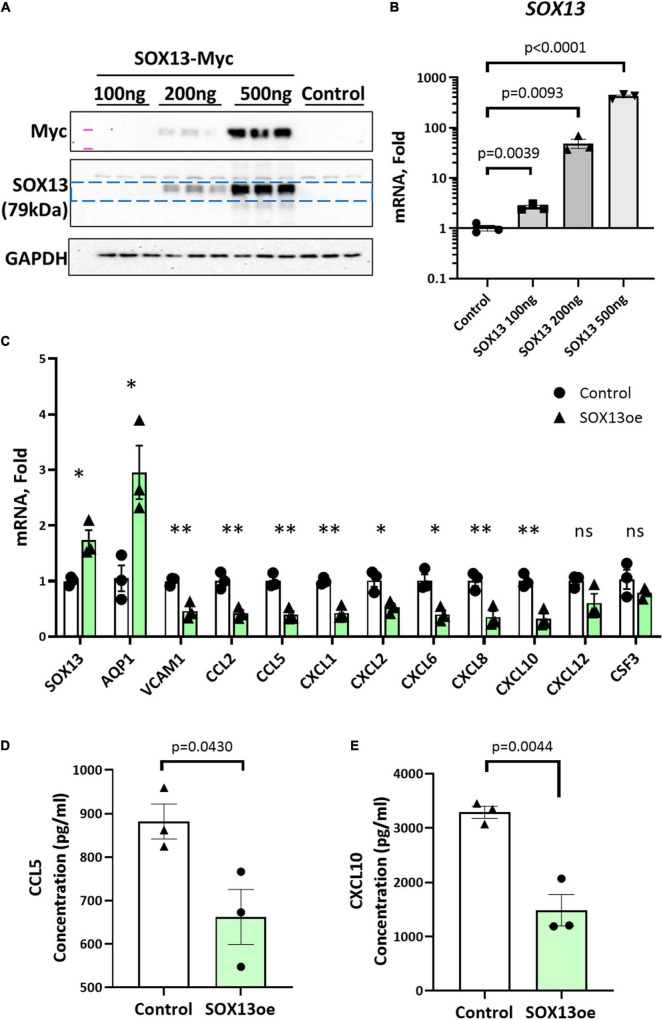
SOX13 plasmid overexpression reduces inflammatory markers and chemokine expression. **(A)** Western blot of whole cell lysate and **(B)** qPCR of SOX13 plasmid concentration curve in static HAECs after 48h compared to GFP control plasmid. **(C)** qPCR analysis of SOX13 targets following 48 h static SOX13 or GFP control overexpression in HAECs at a concentration of 200 ng, using SOX13 and AQP1 as positive controls. *n* = 3, Mean±SEM are shown. *p ≤ 0.05, **p ≤ 0.01 as determined by student’s *t*-test. **(D)** ELISA quantification of CCL5 and **(E)** CXCL10 in the conditioned media from static HAECs, 48 h following SOX13 or control plasmid transfection. Mean±SEM are shown, *n* = 3, *p*-values determined by student’s *t*-test.

Next, we tested whether overexpression of SOX13 can prevent pro-inflammatory gene expression. Transfection with SOX13 overexpression plasmid in HAECs induced *SOX13* and *AQP1* mRNA expression as expected (Figures 7A–C). We found that SOX13 overexpression decreased the expression of the chemokines and cytokines, including *CCL5* and *CXCL10* (Figure 7C). We further validated that the protein levels of CCL5 and CXCL10 were decreased in the CM by SOX13 overexpression compared to the RFP control (Figures 7D,E). Together, these results demonstrated that SOX13 knockdown strongly induced, while overexpression reduced cytokine and chemokine expression in HAECs.

### SOX13 knockdown increases endothelial inflammation *via* CCL5

**FIGURE 8:**
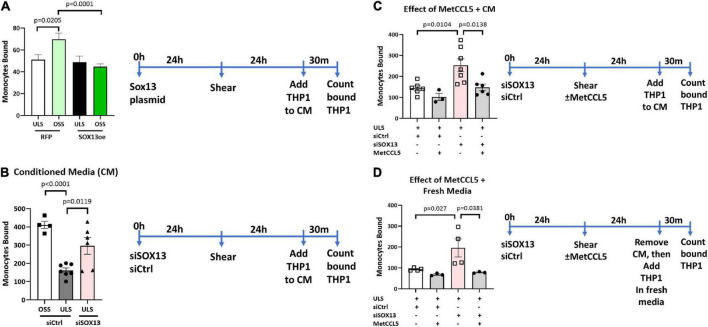
Monocyte adhesion induced by siSOX13 is prevented by treating HAECs with MetCCL5. **(A)** HAECs overexpressing SOX13 or RFP control were exposed to OSS or ULS for another 24 h. Following shear, THP1 monocytes were added to the conditioned media (CM), and adhered monocytes were counted. **(B)** HAECs treated with siSOX13 or siCtrl were exposed to OSS or ULS for another 24 h. Following shear, THP1 monocytes were added to the CM, and adhered monocytes were counted. **(C,D)** HAECs treated with siSOX13 or siCtrl were exposed to OSS or ULS in the presence or absence of MetCCL5 for another 24 h. Following shear, THP1 monocytes were added to the CM, and adhered monocytes were counted **(C)**. In panel **(D)**, following shear, CM was removed, HAECs were washed with fresh medium, THP1 monocytes were added to the CM, and adhered monocytes were counted. Mean±SEM are shown, *n* = 3 to 7, *p*-values determined by one-way ANOVA.

To functionally test the SOX13 effect on EC inflammation, we performed THP1 monocyte adhesion assays using HAECs treated with siSOX13, SOX13 overexpression, and shear. First, we found that overexpression of SOX13 reduced the OSS-dependent increase in monocyte adhesion to HAECs (Figure 8A), demonstrating the role of SOX13 in the OSS condition. Next, we found that siSOX13 increased monocyte adhesion to HAECs under the ULS condition (Figure 8B). Since CCL5 was the most strongly induced chemokine by siSOX13, we used the specific CCL5 antagonist MetCCL5 to test if it can prevent siSOX13-induced monocyte adhesion. First, MetCCL5 was added to the media during ULS exposure for 1 day to inhibit CCL5 acting on ECs. Following the shear, monocytes were added to the CM, containing any residual amount of MetCCL5 following the 24h shear, and the number of adhered monocytes was quantified. In this condition, MetCCL5 treatment prevented monocyte adhesion to HAECs induced by siSOX13 in the ULS condition (Figure 8C). MetCCL5 serves as a specific CCL5 antagonist by binding to the CCL5 receptor on monocytes, leading to inhibition of atherosclerosis in mouse models (54, 55), but its effect on ECs is unknown. Therefore, we tested whether the inhibitory effect of MetCCL5 on monocyte adhesion (Figure 8C) was mediated at the level of ECs. To this end, we added MetCCL5 to the HAECs during ULS for 1 day. Then the CM was removed, HAECs were washed and replaced with fresh media containing THP1 monocytes, and adhered monocytes were counted. MetCCL5 still prevented monocyte adhesion to HAECs treated with siSOX13 and ULS (Figure 8D). These results suggest that SOX13 knockdown induced chemokine expression, including CCL5, which increased monocyte adhesion to ECs. Our results show that MetCCL5 prevented monocyte adhesion to HAECs by directly blocking the CCL5 effect on HAECs.

## Discussion

Here, we identified novel FSTFs, including SOX13, in mouse artery ECs by re-analyzing the *in vivo* gene array, scRNAseq, and scATACseq datasets and validated them in HAECs *in vitro*. We further showed that SOX13 is induced by s-flow or ULS and decreased by d-flow or OSS. While the SOX13 knockdown dramatically induced expression of pro-inflammatory cytokines and chemokines, including CCL5 and CXCL10, SOX13 overexpression caused anti-inflammatory responses. Importantly, the CCL5 antagonist MetCCL5 prevented monocyte adhesion induced by SOX13 knockdown under shear conditions. These results reveal that SOX13, induced by s-flow but reduced by d-flow, is a potent anti-inflammatory FSTF, which represses pro-inflammatory chemokine and cytokine expression in ECs.

Several FSTFs, including KLF2/4, have been previously identified in cultured ECs, including human umbilical vein ECs (HUVECs). While these FSTFs have been critical to understanding flow-sensitive gene regulation, endothelial function, and vascular pathophysiology, some FSTFs may have not been identified because they were lost or dysregulated in ECs under *in vitro* culture. It was estimated that approximately 45% of all flow-sensitive genes are dysregulated or lost in cultured ECs compared to *in vivo* ECs (17, 56, 57). It is possible that those lost or dysregulated genes during culture are highly flow-sensitive ones, including FSTFs. Therefore, we hypothesized that we could discover novel FSTFs by examining *in vivo* gene array and scRNAseq datasets obtained from our PCL studies. Indeed, our present study revealed 12 FSTFs *in vivo*. We determined that 8 of these mouse FSTFs (*KLF2, KLF4, SOX4, ZBTB46, SOX13, SIX2, NFIL3*, and *CEBPβ*) were conserved in human ECs (HAECs) and showed consistent flow-sensitivity. KLF2/4 are best characterized FSTFs (14, 43), while SOX4 and ZBTB46 were also reported as FSTFs (44, 45), confirming the validity of our approach. *SOX13, SIX2, NFIL3*, and *CEBPβ* are potentially novel FSTFs identified in this study. Interestingly, our scRNAseq data analysis showed that SOX13 is preferentially expressed in ECs but not in other arterial wall cell types (smooth muscle cells, immune cells, and fibroblasts). Further, our scATACseq analysis showed that chromatin accessibility of the SOX13 gene, including the promoter region, was highly accessible (indicating a high gene transcriptional activity) under the s-flow condition compared to the d-flow condition in mouse carotid arteries *in vivo*. This indicates that flow regulates SOX13 at the transcriptional level. At the protein level, SOX13 protein is found predominantly in the nucleus. *In vivo*, Sox13 immunofluorescence staining indicates higher intensity in the s-flow-exposed greater curvature of the aortic arch compared to the d-flow-exposed lesser curvature. Also, SOX13 function in flow-dependent endothelial biology was unknown. Based on these, we decided to characterize the role of SOX13 as a novel FSTF.

SOX13 plays an important role in arterial development, T-cell differentiation, and cell fate determination (33, 40, 58). Dysregulation of SOX13 is involved in cancer development (35, 38, 59–61), but its role in atherosclerosis and flow-dependent function in adult ECs was unknown. SOX13 can act as a TF that directly binds to its DNA binding motif (47) or as a cofactor binding to other TFs such as T-Cell Factor1 (TCF1) to indirectly regulate gene transcription (53). To determine how SOX13 induced by ULS regulates endothelial function, we performed the RNAseq study in HAECs treated with siSOX13 under the ULS condition compared to the OSS condition. From the study, we identified two distinct classes of SOX13 target genes: (1) 40 OSS-induced genes that were also upregulated by siSOX13 in the ULS condition (Figure 4H) and (2) 94 downregulated genes by OSS or siSOX13 (Figure 4G). Interestingly, the 40 OSS- or siSOX13-induced genes (including cytokines and chemokines) lacked the SOX13 DNA binding motifs, whereas 70 of the 94 downregulated genes (including developmental genes and the known target *AQP1*) (39, 48, 62–64) contained the SOX13 binding motifs as determined by a MEME motif analysis (65) and comparison to a SOX13 ChIPseq dataset (46). These results suggest that SOX13 regulates its gene targets through direct and indirect mechanisms.

It was particularly interesting that many of the 40 OSS- and siSOX13-induced genes were pro-inflammatory cytokines and chemokines, a novel finding with a potential therapeutic implication. Of those, we validated the dramatic upregulation of CCL5 and CXCL10 by qPCR and ELISA induced by siSOX13 in the ULS condition. In contrast, SOX13 overexpression significantly reduced the expression of CCL5 and CXCL10 mRNA and protein. CCL5 (C–C motif ligand 5, also known as RANTES) and CXCL10 (C–X–C motif chemokine ligand 10) are two well-known cytokines that promote monocyte recruitment and macrophage maturation to atherosclerotic plaques (66–69). Increased serum levels of CCL5 and CXCL10 are associated with human atherosclerotic plaque progression and stability, and the inhibition of either cytokine reduced atherosclerosis in animal models (70, 71).

CXCL10 is a specific T-cell effector and is important in recruiting T-cells to atherosclerotic lesions (70). Interestingly, treatment with CXCL10 neutralizing antibody reduced the size and stability of atherosclerotic plaques in the carotid cuff model of atherosclerosis in ApoE^–/–^ mice (70). CCL5 is found on EC surfaces deposited by activated platelets and produced by other arterial cells, such as smooth muscle cells and macrophages, but the role of ECs as a source of CCL5 is unclear (71). CCL5 recruits monocytes to inflamed endothelium in early atherosclerotic lesions (71). The CCL5 antagonist MetCCL5 inhibits monocyte binding to ECs and reduces mouse atherosclerotic plaque development (54, 55, 72). These literatures demonstrate that both CXCL10 and CCL5 play critical roles in atherosclerosis development, and their inhibition is an effective anti-atherogenic approach.

The present study demonstrated that CXCL10 and CCL5 are regulated by shear stress in a SOX13-dependent manner and that Sox13 appears to play a dominant repressor role in flow-dependent chemokine expression. Our scRNAseq data analysis showed that *CCL5 and CXCL10* expression was upregulated in a time-dependent manner by d-flow. CCL5 expression was increased by 2- and 15-fold at 2 days (E5 vs. E2) and 2 weeks (E8 vs. E2) post-PCL, respectively (Supplementary Figure 1A). CXCL10 expression was increased only in the 2-week, but not in 2-day, post-PCL time point by 3.5-fold (Supplementary Figure 1B). In addition, the gene array data analysis showed a similar chronic flow-dependent response with a significant increase only at the 2-week post-PCL time point, increasing 24-fold for CCL5 and 19-fold for CXCL10 expression, but no significant change at 12, 24, and 48 h time points (Supplementary Figures 1C,D). The mechanism for this relatively slow, chronic d-flow-dependent induction of these cytokines is unclear, but may be related to a gradual loss of SOX13 over time below a threshold level, contributing to an overall endothelial cellular status change. In support of this notion, we recently showed that chronic d-flow induces endothelial reprogramming, including endothelial to mesenchymal transition (EndMT) and endothelial to immune cell-like transition (EndICLT), taking 2 weeks post-PCL (13). Interestingly, we found that *CCL5 and CXCL10* expression did not show a significant flow-dependent response in HAECs by LSS or OSS conducted over a 24 h period. However, *CCL5 and CXCL10* expression was dramatically induced if SOX13 was knockdown by siSOX13. The lack of flow sensitivity of the CCL5 and CXCL10 in the short-term *in vitro* experiment agrees with the *in vivo* data (Supplementary Figure 1). Due to a technical limitation of exposing HAECs to shear *in vitro* for 2 weeks, for an extended period, we did not test the shear response of CCL5 and CXCL10 under a similar chronic condition. Taken together, we propose that d-flow gradually reduces SOX13 expression over time below a threshold level, triggering strong CCL5 and CXCL10 expression through an indirect mechanism in ECs. Interestingly, neither CCL5 nor CXCL10 contains the SOX13 DNA binding motif in the promoter regions. Therefore, we hypothesize that SOX13 plays a dominant repressor role. Even relatively small amounts of SOX13 may bind to another TF, such as NF-κB, preventing its binding and activation of CCL5 transcription. However, under the d-flow or SOX13 knockdown conditions, the SOX13-bound TF such as NF-κB could be released from SOX13 and bind to the CCL5 promoter, increasing its transcription. Interestingly, CCL5 and CXCL10 are known transcriptional targets of NF-κB family members (73). It would be interesting to test whether the loss of SOX13 would activate the NF-kB pathway leading to chemokine induction.

We showed that loss of SOX13 induced monocyte adhesion to ECs in the CCL5-dependent manner. We found that siSOX13 dramatically induced CCL5 protein release by HAECs, even under ULS conditions, as measured by ELISA in the conditioned media. The CCL5 antagonist, MetCCL5, is known to bind to the CCL5 receptors on monocytes, preventing its activation (55). Our study tested if MetCCL5 could inhibit monocyte adhesion by blocking the CCL5 receptors on HAECs (Figure 8). Our result suggests that not only monocytes but also ECs mediate the anti-inflammatory effect, a key anti-atherogenic mechanism, of MetCCL5.

In conclusion, we found several novel FSTFs from *in vivo* mouse artery ECs that are conserved in cultured human aortic ECs. Of these, we show that SOX13 is increased under s-flow conditions, repressing the expression of pro-inflammatory cytokines CCL5 and CXCL10. Loss of SOX13 by d-flow or siRNA induces endothelial inflammation by increasing CCL5 and CXCL10 expression, and MetCCL5 prevents the pro-inflammatory effects. SOX13, CCL5, and CXCL10 are potential atherogenic therapeutic targets.

## Materials and methods

### Mice and partial carotid ligation surgery

Eight-week-old C57Bl/6 male mice (Jackson lab) were maintained and cared for in accordance with the National Institutes of Health (NIH) guidelines in our AAALAC-accredited experimental animal facility at Emory University under a controlled environment (21 ± 2°C, 50 ± 10% relative humidity, and a 12 h light: 12 h dark cycle with lights on at 0700h EST). All mouse studies were approved by the Institutional Animal Care and Use Committee at Emory University and were in accordance with the established guidelines and regulations consistent with federal assurance. PCL surgery was performed under anesthesia by ligating 3 of 4 caudal branches of LCA (left external carotid, internal carotid, and occipital artery) using 6–0 silk suture, leaving the superior thyroid artery patent. The presence of low and oscillatory shear was confirmed by ultrasonography as we previously described (32, 74).

### *In vivo* gene array dataset, single-cell RNA sequencing, and single-cell assay for transposase-accessible chromatin with high-throughput sequencing datasets using the mouse partial carotid ligation study

The gene array data (GSE182291) used in this study was previously reported by us (12). Briefly, endothelial-enriched RNAs obtained from the LCAs and RCAs, following the PCL surgery at 12 h, 24 h, 48 h, and 2 weeks were subjected to whole-genome microarray analysis using the Affymetrix HT_MG-430_PM. For this study, 10 week-old male C57/BL6 mice from Jackson Lab (*n* = 5 each) were used.

The scRNAseq data set (Bioproject # PRJNA646233) and scATACseq data set (Bioproject # PRJNA646233) used in this study were previously reported by us (13). Briefly, intraluminally obtained single cells or single nuclei obtained from the LCAs and RCAs, following PCL at 2 days or 2 weeks, were used for scRNAseq and scATACseq. For this study, 10-week-old male C57/BL6 mice from Jackson Lab (*n* = 10 each for scRNAseq and *n* = 12 each for scATACseq) were used. The sequencing results were analyzed for gene expression analysis by violoin plot and chromatin accessibility assay at the genes of interest using the R-packages as we described (13). Gene ontology analysis and functional pathway analysis Gene ontology analysis was performed using PANTHER.

### Human aortic endothelial cells

Human aortic endothelial cells obtained from (Cell Applications Lot#: 2463) were cultured in a complete medium (MCDB 131, 10% FBS, 1% Pen-Strep, 1% L-glutamine, 1% ECGS, 15 ng/mL IGF-1, 1 mg/mL hydrocortisone, 50 mg/mL ascorbic acid, 5 ng/mL VEGF, 5 ng/mL EGF, 5 ng/mL FGF) (13, 75). The medium was refreshed every 3 days and cells were used through passage 7.

### Cone and plate viscometer

For *in vitro* flow experiments, we exposed confluent cells to steady unidirectional flow (*s-flow*, 15 dyn/cm^2^) or bidirectional oscillatory flow (*d-flow*, +5/-4 dyn/cm^2^ at 1 Hz) to mimic *in vivo* flow profiles using the cone-and-plate viscometer for 24- or 48-h experiments (75). In brief, a cone controlled by a stepping motor is placed in a standard 10 cm tissue culture dish containing confluent HAEC monolayer. The cone was rotated unidirectionally or bidirectionally at different velocities to generate *s-flow* and *d-flow* conditions, respectively. Under these conditions, *s-flow* induces anti-inflammatory KLF2 (Krüppel-like factor 2) expression and *d-flow* generates inflammatory signaling at levels resembling those observed *in vivo* (76–78).

### RNA extraction from cells for quantitative RT-PCR and RNAseq

For RNA collection, cells were lysed with Qiazol and total RNA was purified using the Qiagen miREasy kit (74004) or Zymo Direct-zol Miniprep kit (R2052) (13, 75). RNA was quantified by Nanodrop and was reverse transcribed for use in a two-step qRT-PCR using the High-Capacity cDNA Reverse Transcription Kit (Applied Biosystems 4368814).

### RNAseq data analyses

For RNAseq, HAECs were collected in Qiazol lysis reagent and processed for RNA isolation using miRNeasy Mini Kit (Qiagen 217004). Following quality checks, library was prepared and RNA sequenced at Novogene (*n* = 4 per group). The data was processed with Partek and R package RSubread from Bioconductor, to identify differentially expressed genes, as described (79). Potential direct targets of SOX13 TF binding motifs were examined with USC Genome Browser and a MEME motif analysis (65) and comparison to a SOX13 ChIPseq dataset (46).

### Quantitative RT-PCR for validation of mRNAs

Quantitative Real-time PCR (qPCR) was performed on selected genes using Brilliant II SYBR Green QPCR Master Mix (Stratagene) with custom-designed primers on a Real-Time PCR System (ABI StepOne Plus) (17). All qPCR results were normalized based on 18S RNA expression in the respective sample. Fold changes were determined using the ΔΔCt method (80). Mouse qPCR primers are detailed in Table 1. Human primer sequences are detailed in Table 2.

**TABLE 1:** Mouse qPCR primer sequences.

Mouse gene	Forward sequence	Reverse sequence
m_Ahr	CTGGTTGTCACAGCAGATGCCT	CGGTCTTCTGTATGGATGAGCTC
m_Akna	AGGACCTGTCTCCTTGCCAGAT	GAGACCTCTTGGGCTTTCCTCA
m_Atoh8	CAACGGAGATCAAAGCCCTGCA	CTTCTGCCCATAGGAGTAGCAC
m_Cebpα	GCAAAGCCAAGAAGTCGGTGGA	CCTTCTGTTGCGTCTCCACGTT
m_Cebpβ	CAACCTGGAGACGCAGCACAAG	GCTTGAACAAGTTCCGCAGGGT
m_Creb3l1	AGCCTTGTGCTTCGTTCTGGTG	CATCGTAGAACAGTAGGCTTCGG
m_Etv5	GCAGGAATACCATGACCCACTG	AGGATGACTGGCAGTTAGGCAC
m_Fos	GGGAATGGTGAAGACCGTGTCA	GCAGCCATCTTATTCCGTTCCC
m_Fosb	ACCTGTCTTCGGTGGACTCCTT	TGGCTGGTTGTGATTGCGGTGA
m_Fosl2	AGGAGGAGAAGCGTCGAATCCG	CCAGACTTCTCCTCTTCCAGCT
m_Foxq1	CAACGAGTACCTCATGGGCAAG	GCATCCAGTAGTTGTCCTTGCC
m_Glis3	GGACCCATTTCACCTCCAGCAA	CAAAGTCGTGGACACCAGAGAC
m_Hey1	CCAACGACATCGTCCCAGGTTT	CTGCTTCTCAAAGGCACTGGGT
m_Klf12	CCTTTCCATAGCCAGAGCAGTAC	TGGCGTCTTGTGCTCTCAATGC
m_Klf15	ACACCAAGAGCAGCCACCTCAA	GCCTTGACAACTCATCTGAGCG
m_Klf2	CACCTAAAGGCGCATCTGCGTA	GTGACCTGTGTGCTTTCGGTAG
m_Klf4	CTATGCAGGCTGTGGCAAAACC	TTGCGGTAGTGCCTGGTCAGTT
m_Maf	AGGAGGTGATCCGACTGAAGCA	TCTCCTGCTTGAGGTGGTCTAC
m_Maff	ACCTGTCGGATGAAGCGCTGAT	TAGCCGCGGTTCTTGAGTGTGC
m_Nfil3	CAGGACTACCAGACATCCAAGG	AGGACACCTCTGACACATCGGA
m_Runx2	CCTGAACTCTGCACCAAGTCCT	TCATCTGGCTCAGATAGGAGGG
m_Six1	AGGTCAGCAACTGGTTTAAGAACC	GAGTTGATTCTGCTTGTTGGAGG
m_Six2	CACGCAAGTCAGCAACTGGTTC	ACTTGCCACTGCCATTGAGCGA
m_Sox13	CCTATTCAGCCCATTCCCTGCA	CTTGGCTGTGAGGTTCAGTGGT
m_Sox4	GATCTCCAAGCGGCTAGGCAAA	GTAGTCAGCCATGTGCTTGAGG
m_Sox8	CGCATCTCCATAACGCAGAGCT	TCTTCCTTCGCCTTGGCTGGTA
m_Tcf23	GCTGGAGCAATCACAGACTGAG	TGACGAAGCGTCTTCACCCGAG
m_Tgif1	CAGATTCTGCGAGACTGGCTGT	CGGGCGTTGATGAACCAGTTAC
m_Zbtb46	CACCGTCACTCACTTGGACA	GCAGCTGACATCACCTCGAT
m_Zbtb7c	GAGAAGCCGTACATGTGCAGCA	CACGAACTTGGCGTTGCAGTGA

**TABLE 2:** Human qPCR primer sequences.

Gene	Forward sequence	Reverse sequence
AHR	GTCGTCTAAGGTGTCTGCTGGA	CGCAAACAAAGCCAACTGAGGTG
AKNA	CTCTGGCAACAGTGAGGTGGAG	GGAGAGACTTCACACTGAGGTAC
AQP1	TATGCGTGCTGGCTACTACCGA	GGTTAATCCCACAGCCAGTGTAG
ATOH8	AGCCTTCGAGGCGCTCAGGAA	TCGGCACTGTAGTCAAGGTCAG
BTG2	GCAGAGGCTTAAGGTCTTCAGC	TGGTTGATGCGAATGCAGCGGT
CCL5	CCTGCTGCTTTGCCTACATTGC	ACACACTTGGCGGTTCTTTCGG
CEBPA	AGGAGGATGAAGCCAAGCAGCT	AGTGCGCGATCTGGAACTGCAG
CEBPβ	AGAAGACCGTGGACAAGCACAG	CTCCAGGACCTTGTGCTGCGT
CREB3L1	GCCTTGTGCTTTGTTCTGGTGC	CCGTCATCGTAGAATAGGAGGC
CSF3	AAGGTCGTGCTGGCATTCTG	AGCTGTGATCAGTGGTTGGG
CX3CL1	ACAGCACCACGGTGTGACGAAA	AACAGCCTGTGCTGTCTCGTCT
CXCL1	AGCTTGCCTCAATCCTGCATCC	TCCTTCAGGAACAGCCACCAGT
CXCL10	GGTGAGAAGAGATGTCTGAATCC	GTCCATCCTTGGAAGCACTGCA
CXCL11	AGCAGTGAAAGTGGCAGAT	TTGGGATTTAGGCATCGT
CXCL2	CATCGAAAAGATGCTGAAAAATG	TTCAGGAACAGCCACCAATA
CXCL3	AAAATCATCGAAAAGATACTGAACAAG	GTAAGGGCAGGGACCAC
CXCL6	GGGAAGCAAGTTTGTCTGGACC	AAACTGCTCCGCTGAAGACTGG
CXCL8/IL8	GAGAGTGATTGAGAGTGGACCAC	CACAACCCTCTGCACCCAGTTT
DHX58	ATGACCACCTGGAGATGCCTGA	CATTGTAGCGCCTCAGGTGAAG
EMCN	GCAAGCACTTCAGCAACCAGCC	GGATCTGCCTTCCAGCACATTC
ETV5	GTGTTGTGCCTGAGAGACTGGA	CGACCTGTCCAGGCAATGAAGT
FAM107A	GCTCATCAAGCCCAAGAAGCTG	TCTGGCTTGCTGTCCACACCAA
FOS	GCCTCTCTTACTACCACTCACC	AGATGGCAGTGACCGTGGGAAT
FOSB	TCTGTCTTCGGTGGACTCCTTC	GTTGCACAAGCCACTGGAGGTC
FOSL2	AAGAGGAGGAGAAGCGTCGCAT	GCTCAGCAATCTCCTTCTGCAG
FOXQ1	CCTACTCGTACATCGCGCTCAT	TCGTTGAGCGAAAGGTTGTGGC
GLIS3	AAGCCAGGTCTCTACAGCATGC	ACTCAAGGTCGTGGACGCCAAA
HEY1	TGTCTGAGCTGAGAAGGCTGGT	TTCAGGTGATCCACGGTCATCTG
HLA-F	GCTGCTGTGATGTGGAGGAAGA	GTATGTTCGTGAGGCACAAGTGC
IFI35	CACGATCAACATGGAGGAGTGC	GGCAGGAAATCCAGTGACCAAC
IFI6	TGATGAGCTGGTCTGCGATCCT	GTAGCCCATCAGGGCACCAATA
IFIT3	CCTGGAATGCTTACGGCAAGCT	GAGCATCTGAGAGTCTGCCCAA
IFITM1	GGCTTCATAGCATTCGCCTACTC	AGATGTTCAGGCACTTGGCGGT
IL1β	CCACAGACCTTCCAGGAGAATG	GTGCAGTTCAGTGATCGTACAGG
IL3	CTTCGAAGGCCAAACCTGGA	ATGGATTGGATGTCGCGTGG
ISG20	ACACGTCCACTGACAGGCTGTT	ATCTTCCACCGAGCTGTGTCCA
KLF12	CCTTTCCATAGCCAGAGCAGTAC	CTGGCGTCTTGTGCTCTCAATAC
KLF15	GTGAGAAGCCCTTCGCCTGCA	ACAGGACACTGGTACGGCTTCA
KLF2	ATGACCACCAACCATTGCAC	ACACCTCTCAGCTGTTTCCA
KLF4	CATCTCAAGGCACACCTGCGAA	TCGGTCGCATTTTTGGCACTGG
Lpar1	GGCTATGTTCGCCAGAGGACTA	GGAGTCCAGCAGATGATAAAGGC
MAF	AGAAGTTGGTGAGCAGCGGCTT	CACTGATGGCTCCAACTTGCGA
MAFF	CTGTCGGACGAGGCGCTGATG	AGCCACGGTTTTTGAGTGTGCG
Map4k4	CAACATCTCGCTCCCCTGTT	CCTGGGCTCAATACTGGTGG
MCP1/CCL2	AGAATCACCAGCAGCAAGTGTCC	TCCTGAACCCACTTCTGCTTGG
MFAP5	GGGTCAATAGTCAACGAGGAGAC	GCCAAGTCATCTGTGGAAGGTG
MX2	AAAAGCAGCCCTGTGAGGCATG	GTGATCTCCAGGCTGATGAGCT
NFIL3	TGGAGAAGACGAGCAACAGGTC	CTTGTGTGGCAAGGCAGAGGAA
OASL	GTGCCTGAAACAGGACTGTTGC	CCTCTGCTCCACTGTCAAGTGG
PTGS2	CGGTGAAACTCTGGCTAGACAG	GCAAACCGTAGATGCTCAGGGA
SIX1	AGGTCAGCAACTGGTTTAAGAACC	GAGGAGAGAGTTGGTTCTGCTTG
SIX2	CACACAGGTCAGCAACTGGTTC	TCATCCTCCGAGCTGCCTAACA
SOX13	CCGAAACAGCAGCCACATCAAG	CTGCTTCTCCTGGTTGGTCATG
SOX4	GACATGCACAACGCCGAGATCT	GTAGTCAGCCATGTGCTTGAGG
SOX8	GACCAGTACCCGCACCTG	GCTTCTCGCTCTCGCTCA
TCF23	CCTCCTCAGGCACTGTGTTT	CTCTGGCCTTCCTCTGTGAC
TCF7L2	GAATCGTCCCAGAGTGATGTCG	TGCACTCAGCTACGACCTTTGC
TGIF1	GGATTGGCTGTATGAGCACCGT	GCCATCCTTTCTCAGCATGTCAG
TXNIP	CAGCAGTGCAAACAGACTTCGG	CTGAGGAAGCTCAAAGCCGAAC
ZBTB46	AGCAGGTGGAAGATGACAGCCG	TGCTGGCTTCGGTGACGGACA
ZBTB7C	GGAGAAGCCATACATGTGCACC	ACGAACTTGGCGTTGCAGTGGA

### Protein assay and western blotting

Cells were collected and lysed in RIPA buffer, and analyzed by western blot as we described (13, 75). Briefly, protein concentration was determined with Pierce BCA assay, and Western blot was conducted using Immobilon Western Chemiluminescent HRP (EMD Millipore) and quantified using the Thermo Fisher iBright Imaging system. Nuclear and cytoplasmic fractionation was performed using HAEC lysates with NE-PER^®^ Nuclear and Cytoplasmic Extraction Reagents (Thermo Fisher Scientific 78833) per the manufacturer’s instruction. Antibodies used are listed in Table 3.

**TABLE 3:** Antibodies used for western blotting and immunostaining.

Antibody	Species	Cat. No.	Company	RRID	Concentration
GAPDH	Rabbit	sc-25778	Santa Cruz Technologies	RRID:AB_10167668	1:2500
Myc-tag	Mouse	2276S	Cell Signaling Technology	RRID:AB_331783	1:1000
SOX13	Rabbit	18902-1-AP	Proteintech	RRID:AB_10642149	1:500
Lamin A/C	Rabbit	2032S	Cell Signaling Technology	RRID:AB_2136278	1:500
α-Tubulin	Rabbit	2144S	Cell Signaling Technology	RRID:AB_2210548	1:1000

### Treatment of human aortic endothelial cells with SOX13 siRNA, overexpression plasmid, or MetCCL5

For siRNA knockdown transfections, Oligofectamine (Thermo Fisher Scientific 12252011) was used as a transfection reagent as we reported (13). The siRNAs were purchased from Integrated DNA Technologies (hs.Ri.SOX13.13.1, hs.Ri.SOX13.13.2 and Negative control: 51-01-14-04). The two SOX13 dsiRNA sequences were pooled and treated at a final concentration of 50nM to achieve a robust knockdown. For plasmid transfections, Lipofectamine 3000 (Thermo Fisher Scientific 15338030) was used as we reported (13). SOX13-myc-DDK-tagged (Origene RC210697) was used with a GFP- or RFP-(for monocyte adhesion assay) expressing plasmid with the same backbone as a control (pCMV backbone from Addgene #11153). MetCCL5 (R&D 335-RM-025) was added to the fresh media at a concentration of 50ng/ml at the beginning of the 24 h shear exposure.

### Monocyte adhesion assay

Monocyte binding was determined using THP-1 monocytes (ATCC TIB-202, RRID:CVCL_0006) as we reported (75). In brief, THP-1 cells (1.5 × 10^5 cells/mL) were labeled with a fluorescent dye 2-,7-bis(carboxyethyl)-5 (6)-carboxyfluorescein-AM (BCECF) (Thermo Fisher Scientific B1150; 1 mg/mL) in serum-free RPMI medium (Thermo Fisher Scientific 11875093) for 30 min at 37°C. After exposure to flow or transfection treatments, BCECF-loaded THP-1 cells were added directly to the ECs to the conditioned medium. In some studies, the CM was removed, HAECs were washed with fresh medium, before adding the THP1 cells. After a 30-min incubation at 37°C under no-flow conditions, unbound monocytes were removed by washing the endothelial dishes 2× with HBSS, and cells with bound monocytes were fixed with 4% Paraformaldehyde (Sigma Aldrich 47608) for 10 min. Bound monocytes were quantified by counting the cells under a fluorescent microscope.

### Statistical analysis

Statistical analyses were performed using GraphPad Prism software. All of the n numbers represent biological replicates. Error bars depict the standard error of means (SEMs). The datasets were analyzed for normality using the Shapiro–Wilk test (*p* < 0.05) and equal variance using the *F*-test (*p* > 0.05). Data that followed a normal distribution and possessed equal variance were analyzed using a two-tailed Student *t*-test or one-way analysis of variance (ANOVA) where appropriate. The statistical analysis for scRNAseq differential gene expression analysis was performed using Partek Genomics Suite, GSA (Gene Specific Analysis) (13). This algorithm is a statistical modeling approach used to test for differential expression of genes or transcripts in Partek Flow. GSA can consider the following response distributions: Normal, Lognormal, Lognormal with shrinkage, Negative Binomial, Poisson, and ANOVA. Moreover, it also performs multivariate analysis to see which factors influence that gene.

## Data availability statement

The datasets presented in this study can be found in online repositories. The names of the repository/repositories and accession number GSE207087 can be found in the article/Supplementary material.

## Ethics statement

The animal study was reviewed and approved by the Emory University IACUC.

## Author contributions

CD, SK, D-WK, and HJ conceptualized and planned the experiments. CD, JJ, AA, CP, YK, NV-R, SK, and D-WK performed the experiments. CD, AA, CP, YK, NV-R, and SK analyzed the results. CD and HJ wrote the first draft and edited the final manuscript with input from all authors. All authors approved the submitted version.
