# Supplementary figures and images for: Sox13 is a novel flow-sensitive transcription factor that prevents inflammation by repressing chemokine expression in endothelial cells
Source: Front Cardiovasc Med. 2022 Sep 30;9:979745. doi: 10.3389/fcvm.2022.979745 (PMC9561411; doi:10.3389/fcvm.2022.979745)

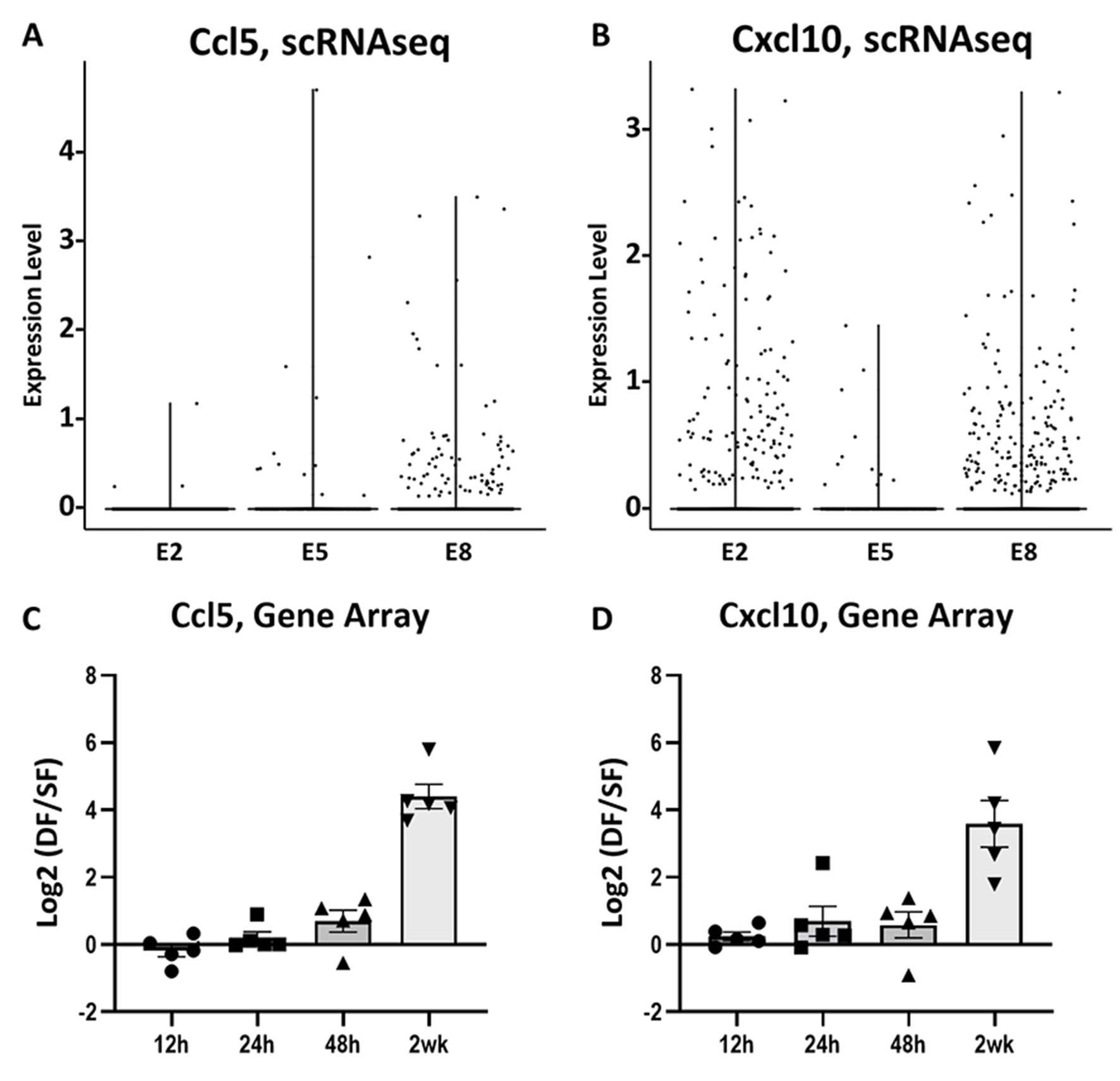

Supplement: Supplementary file 2 [file Image_1.tif]

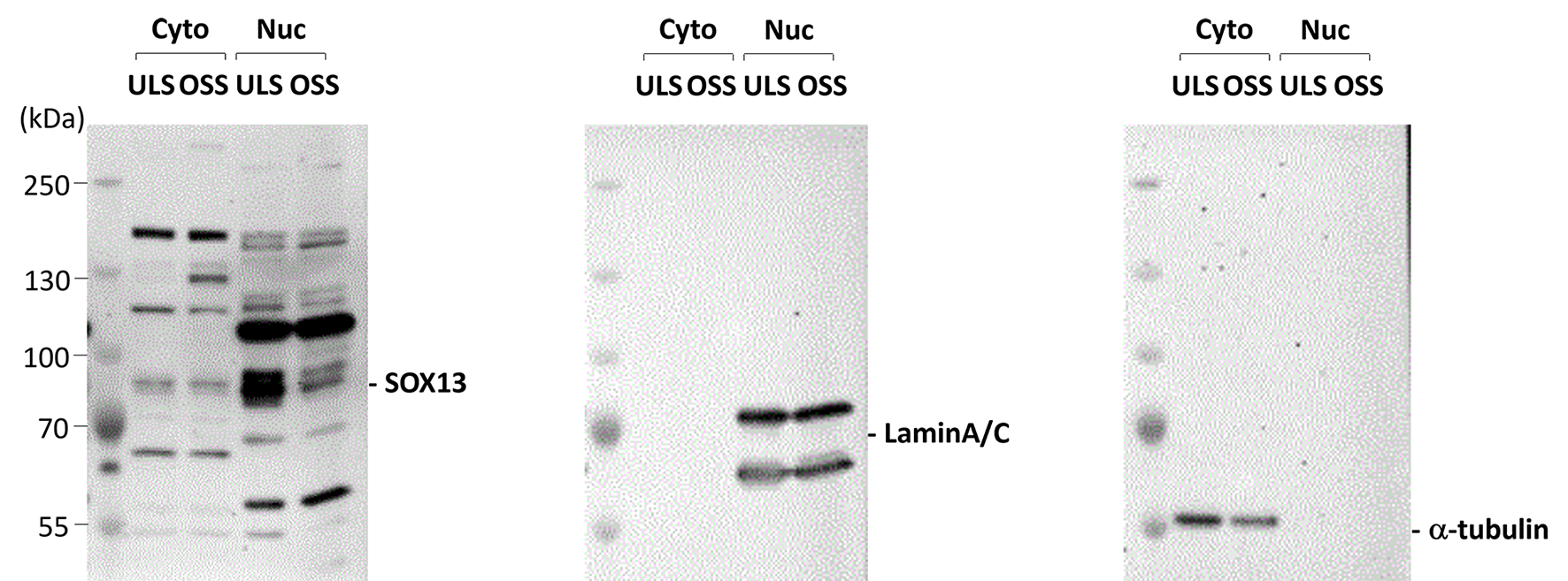

Supplement: Supplementary file 3 [file Image_2.tif]

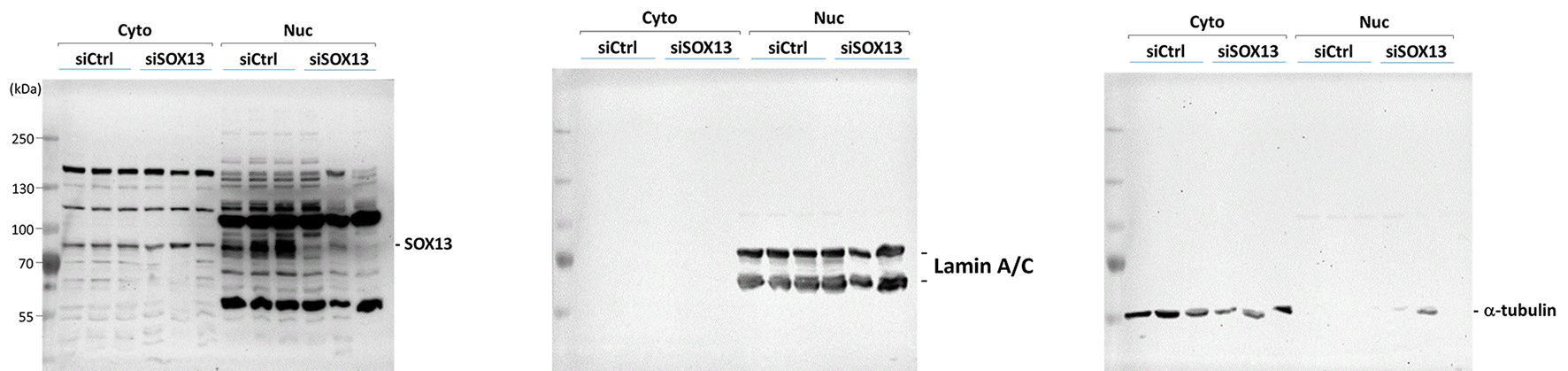

Supplement: Supplementary file 4 [file Image_3.tif]

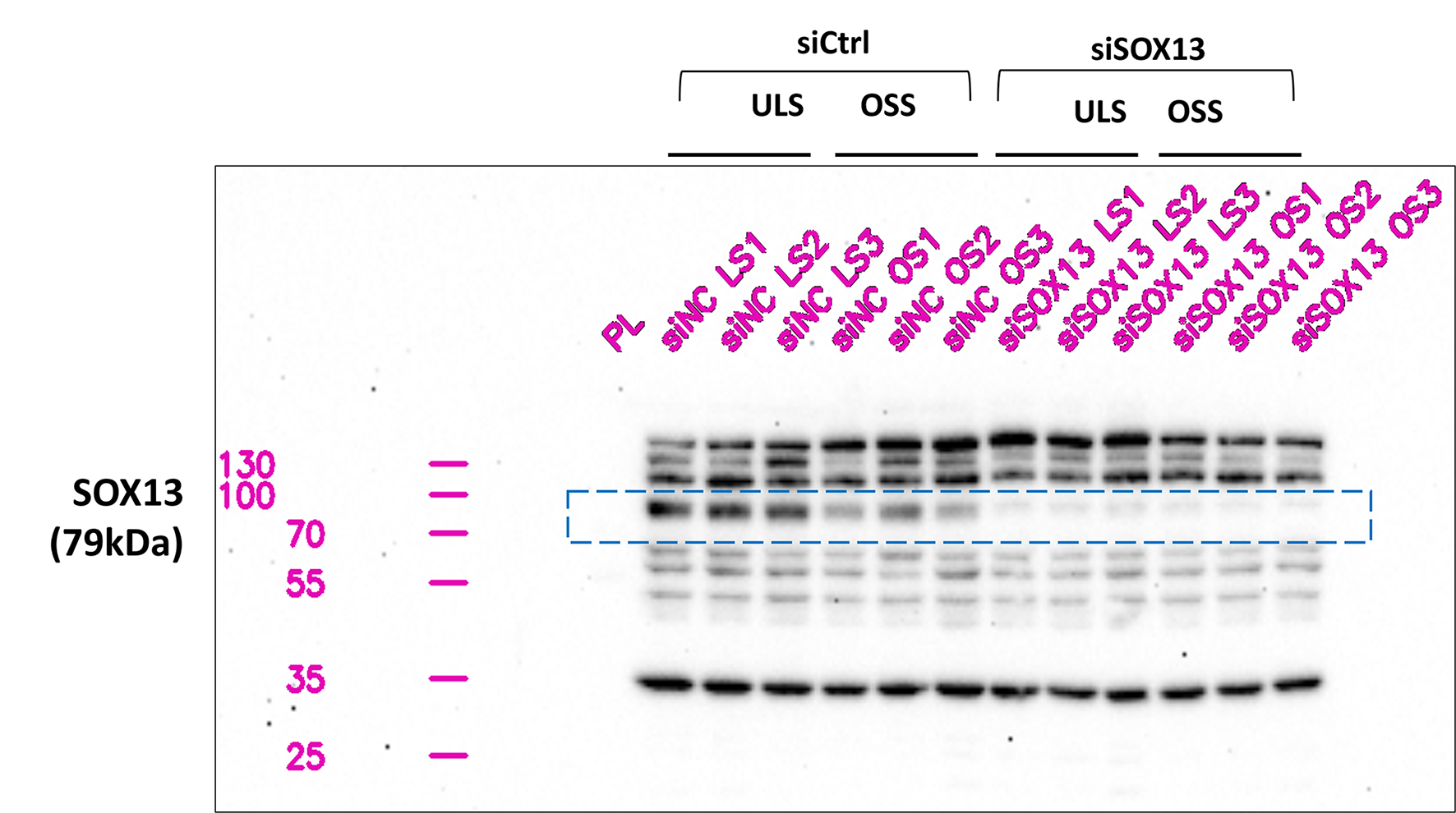

Supplement: Supplementary file 5 [file Image_4.tif]

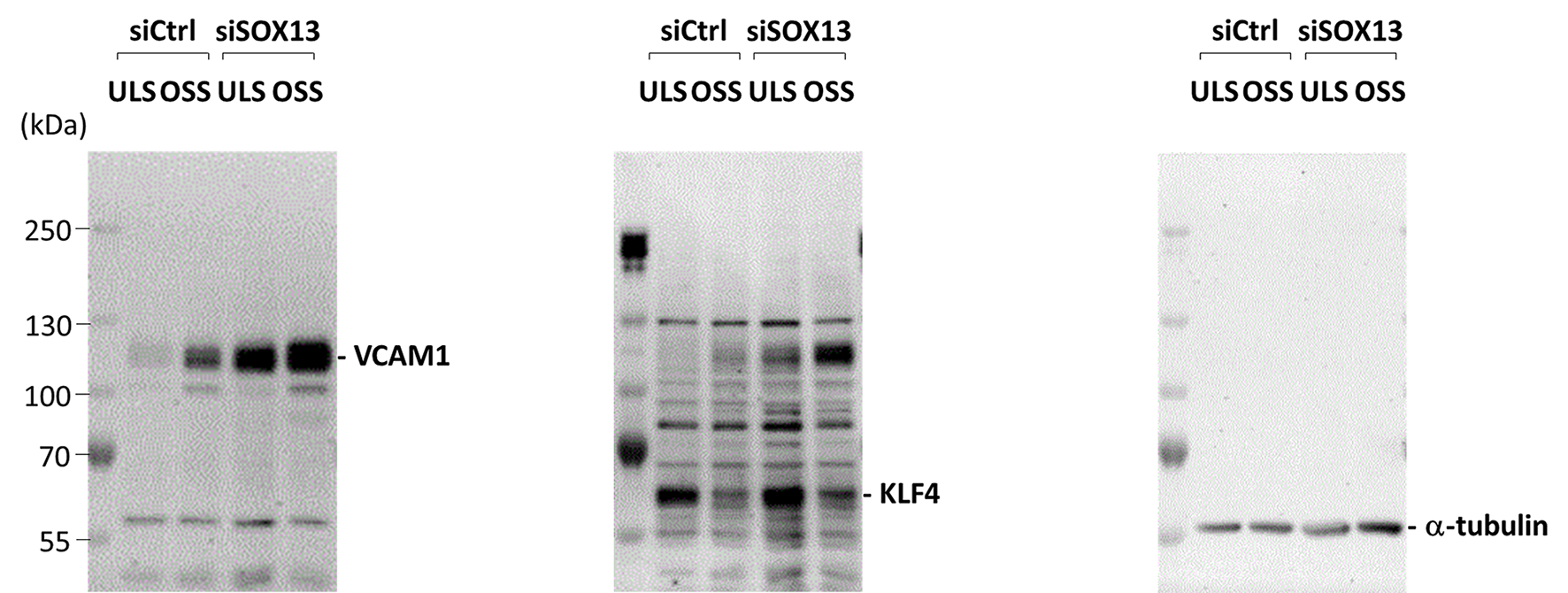

Supplement: Supplementary file 6 [file Image_5.tif]
